# Food Parenting Practices Promoted by Childcare and Primary Healthcare Centers in Chile: What Influences Do These Practices Have on Parents? A Qualitative Study

**DOI:** 10.3390/children10121875

**Published:** 2023-11-29

**Authors:** Paulina Molina, María José Coloma, Patricia Gálvez, María José Stecher, Marcela Vizcarra, Andiara Schwingel

**Affiliations:** 1 Departamento de Nutrición, Facultad de Medicina, Universidad de Chile, Independencia, Región Metropolitana, Santiago 8380453, Chile; paumolina@uchile.cl (P.M.); pagalvez@uchile.cl (P.G.); 2 Department of Viceral Surgery and Medicine, Inselspital, University of Bern, 3010 Bern, Switzerland; mariajose.colomacea@insel.ch; 3 Junta Nacional de Jardines Infantiles, JUNJI, Santiago 7500184, Chile; mstecher@integra.cl; 4 Centro de Investigación del Comportamiento Alimentario, Escuela de Nutrición y Dietética, Facultad de Farmacia, Universidad de Valparaíso, Valparaíso 2360102, Chile; 5 Department of Kinesiology and Community Health, University of Illinois Urbana-Champaign, Fourth St., Champaign, IL 61820, USA; andiara@illinois.edu

**Keywords:** preschool, community organization, childcare center, healthcare center, food parenting practices

## Abstract

Chile, like many other countries in the world, is experiencing a high prevalence of childhood overweight. Among the factors influencing children’s eating behaviors are the food parenting practices promoted by community organizations. More information is needed on the influences of these recommendations on the parenting practices of parents of preschoolers. This study examined what types of food parenting practices are promoted in childcare and primary healthcare centers and how these recommendations influence subsequent parental behavior. Interviews using photo-elicitation were conducted with 25 parents of Chilean preschoolers. The interviews were analyzed using inductive thematic analysis. Five themes were identified to describe food parenting practices promoted by community organizations and the influences that these practices had on parents of preschoolers. Healthcare centers were found to educate parents and provided a structured feeding. However, parents found their advice regarding dietary restriction challenging to follow. Childcare centers also provided information about healthy diet, food monitoring, and diversifying the child’s diet in a way that was perceived as adequate. While childcare centers encouraged structured and autonomous food parenting practices in a positive and supportive manner, healthcare centers tended to provide more restrictive guidance that posed challenges for parents struggling with preschool nutritional care.

## 1. Introduction

Worldwide, overweight affects 5.7% of children under five years of age [1]. In Chile, overweight and obesity impacts 34.6% of children under six years old that are served by the public health system [2], while more than 58% of children between five and six years old who attend public schools live with overweight or obesity [3]. In addition to diet and physical activity, childhood overweight may be due to biological, familial, social, and environmental factors [4]. At the national level, obesity affects a greater proportion of children who are socioeconomically vulnerable, those who belong to an indigenous people, and those who live in rural areas [5]. In preschoolers, a high consumption of ultra-processed foods is associated with overweight and obesity [6,7,8]. Due to the sustained increase in overweight and obesity in infancy and their associated consequences, preventing and addressing this increase is an urgent priority [9].

Attention to overweight and obesity is especially important for preschool children, as they greatly depend on both family and social environments for the development of healthy eating habits [10]. Because overweight and obesity in children are associated with child eating behaviors, it is important to examine the social context in which these eating behaviors emerge. Special attention has been paid to environmental factors that are part of the preschool environment, such as the role of food parenting practices, and the feeding practices promoted by community organizations [11].

The dynamics generated in families, parents, and children at the time of feeding can facilitate the acquisition of healthy eating habits through the promotion of positive parenting practices and the adjustment of these practices to the child’s environment and characteristics [12,13]. Food parenting recommendations provide guidance regarding what, how much, where, and how a child eats [14]. It has been reported that the way a child is fed may be as important as the type and amount of foods a child is given [15]. Food parenting practices have been classified into three main categories: (i) coercive control, (ii) structure, and (iii) autonomy support [14]. The food parenting practices in the coercive control category are those in which adults try to dominate, pressure, or impose the adult’s wishes or goals without significantly considering the child’s needs (for instance, pressuring the child to eat or excessive food restriction) [14]. The structure category refers to the food parenting practices in which adults establish rules and limits to promote children learning about healthy eating (e.g., rules about eating unhealthy foods). The autonomy support category represents practices that encourage independence of the child to eat adequately according to the child’s development. The autonomy support category encourages self-regulation among children (e.g., nutrition education) [14]. It has been suggested that promoting feeding practices in both categories, structure (e.g., encouragement, praise, nutrition education, modeling, and food availability) and autonomy support, is associated with better diet quality and eating habits, leading to better eating behaviors in children [14,16]. In contrast, coercive practices (e.g., restriction, pressure to eat, and food bribes) have been associated with poorer dietary quality and eating habits [17,18,19]. Therefore, food parenting practices in these two categories align with the recommendations of responsive feeding, which involves a reciprocal active feeding interaction between the caregiver and the child in a nurturing environment [20]. This approach promotes autonomous eating and meets the child’s needs regarding hunger and satiety (physiological needs) as well as development (social, emotional, and cognitive dimensions) [20].

In addition to parents and family, other social contexts may be determinants of child’s weight status and weight-related behaviors [21]. In terms of nutrition and promoting healthy living during the first years of life, childcare centers are the most powerful context after the home [22]. Childcare centers determine food choices and the amount of food consumed by the child [21]. In Chile, public childcare services involve care and early education for preschool children from 70% of the most vulnerable families [23]. The two largest networks of childcare centers in the country are the National Board of Childcare Centers (Junta Nacional de Jardines Infantiles, JUNJI) and Fundación Integra [24]. The vision of JUNJI and Fundación Integra encompasses promoting all dimensions of development (physical, cognitive, emotional, and social) [25] through providing early education and holistic wellness in their children, prioritizing families in vulnerable socio-economic conditions [26,27]. The food provided by these childcare centers to preschoolers is regulated by the School Feeding Program [23]. Thus, food is distributed according to national food guidelines and the nutritional requirements of children according to their stage of development [23].

Likewise, primary healthcare centers, through child health checks, are key scenarios for promoting healthy behaviors for children and their families [28]. Preventing and treating childhood obesity with a focus on primary healthcare can be a cost-effective and efficient way to reduce the impact of obesity in this population [29], for example, through child weight monitoring to provide guidance that includes simple and easy-to-follow instructions for caregivers [30]. The Chilean public health system provides services to 79.2% of Chilean children [3], and child health controls in the primary healthcare network are carried out by multiple professionals trained to coordinate preventive actions and promote healthy eating habits, such as doctors, nurses, and nutritionists/dietitians, among others [2,4]. These actions start from gestation, through the control of weight gain in the pregnant mother and the promotion of breastfeeding, and continue with periodic health checks from birth until the child is 9 years old. Additionally, special programs may include sports workshops, education in healthy eating, school gardens, and other participatory activities with parents [2,4].

Although community organizations such as childcare centers and primary healthcare centers are important for preventing or treating childhood obesity, in Chile, to date, most research has been conducted on school settings [31]. A few studies have addressed food parenting practices, but only to identify them in parents and evaluate associations with anthropometric measures of children and parents [32]. Thus, to our knowledge, the food parenting practices promoted by childcare centers and primary healthcare centers and their effect on parents have not been reported [21]. Despite the wide coverage of these organizations in Chile, there is limited research on their influence on food parenting practices and how parents perceive recommendations regarding these practices. Therefore, the objective of this study was to examine what types of food parenting practices are promoted in childcare and primary healthcare centers and how the recommendations of these practices influence parents. Understanding more about these family–community organization interactions can give us valuable information about the challenges and opportunities facing healthcare and childcare centers when supporting families from disadvantaged backgrounds to prevent and treat childhood obesity.

## 2. Method

We used the Consolidated Criteria for Reporting Qualitative Studies (COREQ) for guiding the report of our study [33].

### 2.1. Research Team Reflexivity

MV conducted the interviews in Spanish and there were no non-participants present during the interviews. MV is a female Chilean nutritionist/dietitian and was a PhD candidate at the time of the study. MV was guided during the study by co-authors AS and PG. There were weekly research group meetings to promote reflection among the nutritionists/dietitians providing nutritional care to young children. MV has experience working in a primary healthcare center in Chile and MJS has experience as a dietitian supervising childcare centers. None of the authors and co-authors had prior relationships with participants of the study. In the study, participants were informed that MV was a PhD candidate in community health. When participants inquired about credentials, MV disclosed her qualification as a nutritionist/dietitian.

### 2.2. Study Design

This is a qualitative research study aimed at exploring food parenting practices promoted at childcare and primary healthcare centers and their effect from the perspective of preschool children’s parents under the pragmatism paradigm. In the pragmatic worldview, a main focus is on applications and how to solve problems (real-world practice-oriented), using different methods and approaches to understand a problem, including subjective and objective knowledge [34,35]. Thus, the pragmatic perspective reflects the lead author’s worldview regarding the work as a nutritionist/dietitian and as a researcher.

The project’s protocol was approved by the Institutional Review Board of the University of Illinois Urbana-Champaign (protocol # 19014) and by the host institution, JUNJI. Written informed consent was obtained and formally recorded from all participants. Assent was obtained from children.

#### 2.2.1. Participant Selection

It was a purposive sample. The inclusion criteria were as follows: being a mother or father or a child’s legal guardian who was in charge of feeding the child after school hours; having a Chilean nationality; and having a child between three and five years old, with no health conditions regarding child growth or development.

Parents and their children were invited through informative fliers on childcare centers’ boards. Moreover, invitation letters and consent forms explaining the study were sent through teachers and teachers’ aides to parents. Parents interested in participating were contacted via phone to verify that they met the inclusion criteria and answer their questions regarding the research before having the first meeting. A meeting was held with the parents who met the inclusion criteria, at which time they had the opportunity to ask questions and address any concerns. Of the 30 parents invited, 25 participants completed the study. One participant could not be contacted again, and four indicated their inability to participate due to time constraints.

#### 2.2.2. Setting

The study was conducted in Santiago, the most densely populated area in Chile and which has high levels of socio-economic inequality [36]. Parents were recruited from 9 childcare centers belonging to the National Board of Childcare Centers. This public organization offers care and early education including a meal program for children, and it prioritizes children from vulnerable backgrounds between 0 and 4 years old [26]. The childcare centers were located in vulnerable areas, defined by a multidimensional poverty index [37].

The interviews were primarily conducted in a room within the childcare centers or at the participants’ home. The participants that completed the research included 24 women and 1 man, with an average age of 32.1 (see Table 1).

#### 2.2.3. Data Collection

Our research team collected data between October 2018 and January 2019. We conducted semi-structured interviews using the photo-elicitation technique [38], consisting of talking about photos previously taken by the participants to promote empowerment and encourage verbal explanation about a topic related to food and nutrition [39]. Interviews with photo-elicitation were used to collect data because they allowed participants to freely express their ideas [40,41].

The interviewer applied the SHOWeD technique [42] to promote an in-depth description of the information, facilitating the conversation with participants through questions such as “What do you see in this picture?”, “What is going on?”, etc. [43]. The photo-elicitation technique was used because it has been shown to be feasible to apply to deepen the study of eating in people of low socioeconomic status [39] and also because it has been used favorably in Chile to deepen the understanding of feeding behavior in women [39] and in parental feeding practices, in a similar sample of parents of low-income Chilean preschoolers [44].

In the first meeting, participants were given instructions about the use of a disposable camera and the ethical considerations about taking photos. They were told that they could take photos of any moment, situation, people, or place relevant to feeding their children or where their children eat to explore how these influence the way parents or guardians feed their children. The participants took photos for 7 to 21 days. The interviews lasted an average of 70 min.

An interview guide was developed by the research team (see complementary material), but the conversation regarding the photos and SHOWeD technique allowed us to collect rich in-depth data as reported in previous studies [39]. The interview guide was discussed by the research team but not pilot-tested.

The interviews were audio-recorded and transcribed verbatim. The lead researcher revised the transcripts for accuracy. Notes were taken during and after the interview to associate information with photos and to summarize the ideas of the participants.

Data saturation was discussed among researchers, and it was reached at the 22nd interview, but a decision was made to interview all 25 participants who agreed to complete the study.

### 2.3. Data Analysis

**Figure 1:**
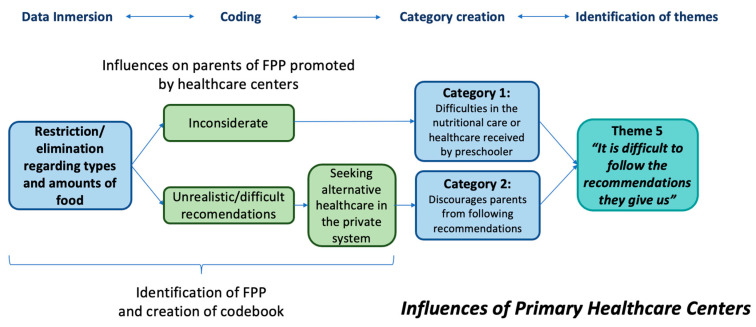
Example of the inductive thematic analysis process used to develop themes related to the effect of recommendations on food parenting practices in primary healthcare centers.

An inductive thematic analysis was conducted. The analysis focused on codes and categories related to the influences of childcare centers and primary healthcare centers in food parenting practices. In this analysis, Vaughn’s [14] framework was used to categorize the food parenting practices (coercive, structure, and autonomy support) promoted by the centers and then to code the effect of those practices on parental perceptions. Codes were developed independently by two team researchers, who created a codebook after discussion about the codes. Later, three team researchers conducted the coding and inductive thematic analysis [45]. The coded information was checked, and categories were developed to generate the themes. An example of the analysis process is found in Figure 1. The software AtlasTi, 8.0.43, was used to organize and analyze the data.

To protect the confidentiality of the participants [46], only the members of the research team had access to the computer with the audio files of the interviews and the database for the thematic analysis; numerical codes were assigned to the transcribed interviews to protect the identity of the participants. The rigor of the research is reflected in the triangulation of the coding process, in which three researchers independently coded the interview data [47]. It is also reflected in the interviewer’s experience in conducting interviews with photo-elicitation [48]. Finally, we analyzed negative cases, and the results were verified by the participants who we were able to contact after the analysis.

## 3. Findings

From the data analysis, 19 codes were identified that related to food parenting practices promoted in childcare and primary healthcare centers and their influences on parents of preschool-age children. Table 2 and Table 3 show a comparison of the main food parenting practices, themes, and representative quotes for both organizations.

### 3.1. Public Childcare Centers

Theme 1. *“They let me know what and how much my child eats”*

Participants stated that teachers and teacher aides gave information about the amounts and types of food their child eats during childcare hours (monitoring) (n = 19/76%). They said that teachers and teacher aides detailed the food type, amount, and eating behaviors, including times when the child eats or does not eat the meals offered by the establishment daily. This provided information generates conversations about food parenting practices between the teachers and teacher aides and parents to help to ensure children’s adequate food intake. Parents reported that two indicators they used to reinforce the child’s eating at home were the information given by teachers and teacher aides and their child’s own communication of feeling hungry.

Theme 2. *“They give the best foods for my child”*

Most parents reported that childcare centers offered a structured environment for children to eat (n = 16; 64%). This structured environment consisted of following: a healthy monthly schedule, establishing mealtimes, and presenting age-appropriate foods. This structure positively influenced the eating practices applied by parents at home by encouraging better habits, incorporating new foods and ways of offering them, and helping to avoid sudden changes in a child’s eating routines, despite the challenges this represented due to the differences with the current family eating routines.

In addition, parents indicated that the childcare centers provided adequate food for their children (n = 12; 48%), emphasizing that these centers offer homemade, healthy, and varied preparations to preschoolers. They also said the childcare centers consider their children’s preferences (e.g., do not drink or eat some food as the child indicates) and particular conditions, such as food allergies or intolerances.

Theme 3. *“They promote a healthy lifestyle for the whole family”*

Some parents (n = 10; 40%) said that childcare centers promote a healthy life by carrying out educational family activities on various food topics. The participants recognized that childcare centers also educate their children regarding a healthy weight, the food warning labels, and the relationship between consuming sweets and the development of cavities. As a result of these activities, the children subsequently identified these foods, particularly those with warning labels, and encouraged their families to decrease their consumption.

Some parents indicated that childcare centers plan entertaining activities and intercultural educational experiences (or theme weeks), including food preparation and tasting. Parents valued these activities because they created opportunities for children to get involved with new foods (n = 5; 20%) and eventually incorporate those foods into the home.

### 3.2. Primary Healthcare Center

Theme 4. *“They educate us on what to feed our child”*

Most participants indicated that healthcare professionals educate families about food and nutrition, including children, during health check-ups (n = 16; 64%). They said that the benefits of healthy eating and foods, weight status, child growth, and consequences of children being overweight were topics that healthcare professionals approached during the health check-ups. Some parents highlighted that healthcare professionals such as nutritionists gave them instructions regarding the schedule to eat, food types, and/or food amounts for the child (n = 10; 40%). A few parents valued this information (n = 5; 20%) because it is a way to give structure for their child to eat. However, other parents considered this information as less valuable because of how the professionals interacted with them during the health check-up, e.g., using merely printed material with restricted foods, general recommendations, or a diet instead of giving practical suggestions about how to implement these recommendations.

Theme 5. *“It is difficult to follow the recommendations they give us”*

Most parents (n = 18; 72%) stated that some of the recommendations they receive in primary healthcare centers are not satisfactory to them. They perceive these recommendations as difficult to implement and unrealistic considering the challenges of feeding a young child. They also claim that professionals at primary healthcare centers do not consider their knowledge about food and nutrition and the strategies they use to feed their children. Some parents (n = 9; 36%) indicated that healthcare professionals usually promote restrictive practices regarding the types of foods their children are allowed to eat and the amount or elimination of some foods in their child’s diet. They further argue that this situation discourages them from following the recommendations and confuses them when making the best decisions to feed their children. The parents stated that restricting or eliminating food from their diet is not feasible for multiple reasons: food restriction or elimination promotes more desire in children to eat those foods; they (parents) prefer to provide foods that the child likes, in reduced amounts, or when they feel the child is hungry; the child consumes all their meals at the childcare center; or their children are seen as having an adequate weight. The inconsistency between the recommendations received and the child’s real needs resulted in parents seeking care in the private health system, to be attended to by professionals responsive to their views on nutrition.

## 4. Discussion

This study examined the types of food parenting practices promoted by two important community organizations for preschool-aged children in Chile. Five themes emerged from the data analysis.

The participants recognized various positive aspects of childcare centers, such as the provision of healthy food, the exposure to various foods, and the improvement in the children’s acceptance of varied and new foods at home. These results coincide with the perceptions of parents from other countries, who have described the childcare center as an organization that not only delivers varied, sufficient, and quality food but also positively influences the child’s diet at home [49,50,51]. Previous studies have also shown that children increase the diversification of foods consumed as a result of greater exposure to these foods in childcare centers [52], probably due to the influence of their peers and the playful eating environment of the childcare center [53]. In Chile, public childcare centers are part of a government program aimed at holistic child development in preschoolers, including the experiences involving food. The national food program offered in these childcare centers regulates the nutritional quality of meals [54]. Accordingly, childcare centers have a key role in child development and build bridges to work with families from a nutritional and developmental perspective.

Parents appreciated that childcare centers implemented food parenting practices to promote structure and autonomy, such as establishing meal routines, nutrition education, and food intake monitoring, all of which are considered beneficial to develop healthy eating habits [7]. In line with our findings, previous studies have supported the role of schools in promoting healthy eating patterns, as they are organizations that bring together a high proportion of children [55] and have tools that can be used to promote better nutrition and, thus, prevent childhood obesity [56]. For example, a recent review revealed that social modeling is useful for improving eating behaviors and preventing overweight in young children [57]. A number of factors have been identified in caregivers that can influence the food parenting practices they apply with children in childcare centers, such as their attitudes, their level of training, and the center’s childcare context [58]. The promotion of structure and autonomy by childcare centers is a practice that characterizes responsive parenting and feeding styles and that has been shown to be effective in promoting healthier food preferences in children [20,59]. Therefore, it is important to continue researching the food parenting practices applied and promoted in childcare centers to encourage responsive feeding, which encourages child self-regulation and autonomy [20] and thus promotes better nutrition, especially when serving/caring for a vulnerable child population [60].

In the context of childcare centers, it has been acknowledged that children’s healthy eating initiatives involving parents reinforce the development of capabilities/abilities in teachers, parents, and children [61]. JUNJI’s childcare centers promote the participation of the family and the implementation of strategies to strengthen communication with parents [62,63]. The emphasis on family involvement may be why parents highlighted the communication flow between them and the teachers as well as the teacher aides, who discussed how the child eats during childcare hours. Due to the closeness the childcare centers have with parents and families, childcare centers seem to be a communication bridge between government policies and the community, which may make these centers the ideal setting for promoting healthy eating behaviors [61,64] and preventing early childhood overweight [65]. This study contributes to understanding aspects that could be promoted in the public childcare environment during interventions targeting overweight Chilean preschoolers.

The healthcare center was another organization relevant for parents who valued the food and nutrition education provided by healthcare professionals. The professionals gave messages about nutrition, amounts, and adequate mealtimes, which allowed some parents to develop the structure to feed their children. However, when this information is given in the context of nutritional counseling, especially for treating overweight children, parents often consider the recommendations as restrictive, unidirectional, and not pertinent to the child’s food preferences. This situation led them to be reluctant to follow the recommendations, which they claimed did not consider their beliefs and expertise about their child and feeding strategies.

The current Chilean Policy in Food and Nutrition establishes humanized nutritional counseling that is person-centered, holistic, and respectful of people’s needs and expectations [66]. In addition, the nutritionist is the main person responsible for the nutritional treatment of children with malnutrition. However, it is the task of all primary healthcare professionals to identify risk factors for malnutrition, monitor weight, and promote healthy eating in child health checkups [2]. Previous studies have evaluated the potential role of health professionals in the comprehensive approach to childhood obesity [67,68], but these professionals face barriers in primary care to fulfill this role [69]. Our study suggests that improving communication between health professionals and families will help to address nutritional issues [69]. This better communication could be achieved by addressing potential barriers to adequately managing childhood overweight, such as lack of knowledge, inadequate training, and the limited time of healthcare professionals [69,70]. Better communication and training of health professionals could improve the understanding and implementation of nutritional practices and food recommendations during the nutritional counseling of children.

The participants stated that the child nutritional counseling received was focused on promoting restrictive food parenting practices and indicating what and how much the child could eat, an approach that was not enough to change their children’s eating habits. Evidence discourages the use of excessive restriction to promote healthy habits [14,71]. On the contrary, the promotion of responsive feeding [20] through the use of various responsive feeding practices (e.g., verbalizing, making eye contact, and not pressuring the child to eat) has been associated with adequate development of dietary habits and nutrition [59]. The parents in our study stated that the nutritional care they received was insensitive to the child’s appetite, characteristics, and reactions when feeding the child. This discrepancy between the information provided by professionals and the parents’ needs was an important limitation of the nutritional counseling, making parents feel rather unwelcome by the health professional.

Although the results of this study show that community organizations promote food parenting practices of both structure and autonomy, it seems that both the time of exposure to food parenting practices and the context in which these practices are promoted are relevant factors for the value assigned by parents to each community organization. On the one hand, the childcare center exposes the children daily and directly to the food parenting practices promoted by the educators during their feeding, generating a feeding context where educators and peers participate, and which has been considered ideal for the development of healthier food preferences [72]. On the other hand, the healthcare center promotes food parenting practices in a more indirect and theoretical manner: first, by increasing the frequency of nutritional counseling, which is sporadic and only increases if the child is diagnosed with deficit or excess malnutrition [2]; and second, by covering the limitations of the Chilean primary healthcare system, such as the lack of continuous training of health professionals and the insufficient economic and human resources to meet the demand [70].

In the context of preschool children’s nutritional counseling, effective communication between health professionals and parents is critical. The lack of consideration of the parents’ opinions, values, and beliefs reflects nutritional care focused on the professional rather than on the user and their context. This research suggests the need to establish communication and a relationship based on trust with parents, which are both principles promoted in the current Family and Community Health Care Model in Chile [73]. Understanding the perspectives and needs of parents when feeding their children could help professionals develop better communication with families, strengthening the bond with them and improving nutritional care. In this sense, the advice of healthcare professionals to incorporate different communication strategies, such as motivational interviewing, could be a promising strategy for treating childhood obesity and promoting healthy child development [74,75].

This study has some limitations. The participants were mostly mothers, so the results may not reflect fathers’ views but mostly maternal ones. In addition, the preschoolers were recruited from public childcare centers in the Metropolitan Region. Therefore, the data do not represent parents of children attending private establishments and living in other regions of the country. Finally, this research focused on studying the food parenting practices promoted by two community organizations characteristic of preschool-aged children in Chile, limiting the understanding of the influence of other relevant community organizations. Further research could broaden the understanding of how health centers, childcare centers, and other community organizations relevant to children’s environments promote diverse food parenting practices, and what impact these food practices have on preschool children’s eating behavior.

Given that this is a qualitative research study, this research study does not attempt to generalize the results to a wider population but rather to grasp parents’ thoughts and experiences in regard to how professionals’ promotion of certain food parenting practices impacted them and their families [76]. Nevertheless, the findings of our research provide evidence about the need for a family-centered approach to promote the healthy development of young children and address highly prevalent nutritional issues in childcare centers and primary healthcare centers in Chile. Similar findings could occur among parents of vulnerable preschool children attending public childcare centers and primary healthcare centers of cities in other developing countries with similar characteristics in Latin America and the Caribbean. For example, in urban areas of Peru, the prevalence of overweight and obesity in children is high [77,78]. Peru, similar to Chile, is a country that has declared objectives aimed at holistic care in early childhood and has primary healthcare centers as part of a holistic family- and community-centered model in primary care [79]. Despite the potential transferability of our findings, it is necessary to develop further research to determine whether similar food parenting practices are promoted by community organizations and the effect these feeding practices have on them because these practices are influenced by culture [80].

### Implication for Practice, Policy, and Future Research

Based on the findings of our study, we suggest developing culturally sensitive guidelines for working with parents of young children during health check-ups and treatment for overweight/obesity. These guidelines should adopt a family-centered approach in which parents feel that they are consulted when making decisions about the overweight/obesity of their child. Likewise, training for healthcare providers to strengthen the food parenting practices that they promote in parents (e.g., to create eating routines) and those that would be beneficial to motivate parents and other caregivers is important [14]. These guidelines and the specific training of healthcare providers can increase adherence to treatment for obesity and attendance to healthcare check-ups by reducing the emphasis on coercive food parenting practices in favor of more structured and autonomous strategies [14].

On the other hand, the participants recognized childcare centers as an environment that encourages healthy eating in children and guides parents mainly through food parenting practices that give structure and promote autonomy in children. Thus, efforts to implement the National Policy of Food and Nutrition could focus on childcare centers for approaching nutrition-related issues. Leveraging the actual food-responsive practices applied in the childcare centers and the positive relations that teachers and teacher aides have with families could be a model for promoting healthy child development.

Additional research into the food parenting practices that healthcare providers recommend in primary healthcare centers and those among teachers and teacher aides in childcare centers can expand the findings of our study. Examining the attitudes of parents toward healthcare professionals in a mixed method study could help to support better practices that are culturally sensitive and centered in families. Also, examining the knowledge of healthcare providers, teachers, and teacher aides regarding responsive feeding from a practical perspective can be beneficial to strengthen the services we already provide to vulnerable families with young children. The knowledge regarding responsive feeding is particularly important given the extensive coverage of the primary healthcare system and the network of childcare centers.

## 5. Conclusions

Both childcare and primary care health centers promote preschoolers’ food parenting practices. In childcare centers, the promotion of structured and autonomy practices predominated through the monitoring, education, diversification, and structuring of the child’s diet, which led parents to value the role of this organization positively. Regarding the health centers, structure and restrictive practices were promoted, and the latter did not match the family expectations to improve their children’s diet. This study shows that the approach of community organizations to promote food parenting practices influences the attitudes of the preschoolers’ families to addressing childhood obesity in vulnerable families in Chile.

## Figures and Tables

**Table 1:** Participants’ socio-demographic and family characteristics.

Characteristics	Total Participants n = 25
Average age and age range (years)	32.1 (21–49)
Education level, n (%)	
Elementary	3 (12)
High school	13 (52)
Vocational	3 (12)
Higher education	6 (24)
Work status, n (%)	
Unemployed	5 (20)
Currently working n (%)	20 (80)
Housing	
Own a house	10 (40)
Rent a house	5 (20)
Live with other family members	10 (40)
Household size (range)	2–14
Type of caregiver, n (%)	
Mother	24 (96)
Father	1 (4)
Family type n (%)	
Two-parent family	20 (80)
One-parent family	6 (24)
Type of health insurance n (%)	
Public	18 (72%)
Private	2 (8%)
Both	5 (20%)

**Table 2:** Comparison of the main food parenting practices promoted by the childcare center and the primary healthcare center.

Category of Food Parenting Practices	Childcare Center	Primary Healthcare Center
Structure	Monitoring (n = 19)	Limited/guided choices (n = 17)
	Teachers/teacher aides give information about child eating behaviors (how much, what foods the child ate or did not eat) or parents ask about child eating behaviors.	Healthcare providers promote the reduction in portion sizes.
	Parents can assess whether they should give more food or can understand why their child eats more or less at home.	Recommend healthier food brands and replace foods for the child with other healthier options.
	Meal and snack routines (n = 16)	Meal and snack routines (n = 10)
	Childcare centers have a schedule for providing meals and snacks. Components of lunch are salad, main course, and dessert. Parents try to keep the meal routines at home (schedule and food order at lunch) but more flexibly. Childcare gives their meal plan weekly to families. Parents can go to the childcare center to feed their children or help teachers to feed the children during mealtimes.	Healthcare providers promote meal/snack routines through the delivery of a meal plan or feeding guidelines that include schedules, meal times, and amounts of food for the child.
	Limited/Guided choices (n = 9)	
	In the childcare center, healthy food is offered, but teachers and teacher aides ask parents about food preferences, allergies, etc.	
	Food availability (n = 9)	
	Childcare centers provide meals with adequate energy. Foods are diverse because they offer fruits, vegetables, legumes, etc. Children are not allowed to bring food categorized as less healthy (high in calories, sodium, and saturated fats, among others, according to nutritional warning labels on food and drink products).	
Autonomy support or promotion	Nutrition education (n = 10)	Nutrition education (n = 16)
	Parents: Childcare centers promote a healthy lifestyle by offering workshops about foods, healthy eating, and obesity.	Healthcare providers educate parents about the benefits of healthy eating, child weight, growth status, and the consequences of excess weight.
	Children: Childcare centers educate children about nutritional warning labels on food and drink products, a healthy weight, and the relationship between sugary food and cavities.	
	Child involvement (n = 5)	
	Teachers/teacher aides prepare mixed fruits and other foods with children. They also encourage children to eat by themselves.	
	Depending on the theme of the week, teachers/teacher aides provide opportunities for children to taste varied food and preparations, including fruits, legumes, vegetables, fish, etc.	
Coercive control	Restriction (n = 2)	Restriction (n = 9)
	Childcare centers suggest avoiding the consumption of unhealthy foods (e.g., with nutritional warning labels on food and drinks).	Parents indicate that a strong and frequent suggestion from healthcare providers is food restriction (type or quantity) of a diverse food such as unhealthy foods.

**Table 3:** Quotes from interviewed participants per identified themes.

Themes	Key Quotes and Parental Practice They Promote	
**Public Childcare Centers**		
Theme 1. *“They let me know what and how much s/he eats”*	Monitoring (structure): *“The childcare center does not force any child to eat, but it does let parents know about it when they pick them up, “you know, [the child] did not eat,” so they give the children something else at home... and the teachers here also say “the child has not eaten anything”, so I know I have to have something for my child back home”* (E16)	Monitoring (structure): *“I ask [the teachers or teacher aides at the childcare center], “Did the child eat?”, and they say “No, they ate this food, and we gave them more milk… they ate half a bread bun more.” We still get home to reinforce food…every day we need to ask the childcare center teachers… they always informed me, every day they told me if the child had had milk, if they’d had lunch, without me asking. “Do not worry, today he ate 100%,” which means the child has eaten all the food…”* (E24)
Theme 2. *“They give the best foods for him/her”*	Meal and snack routines (structure): *“[Related to the mother learning about the child’s feeding thanks to the childcare center… it was about eliminating some foods not needed, I mean, maybe add dessert or something like that* [*like in the childcare center*]*, because before I gave the child the meal and a juice, but here they are used to giving them the dessert and the salad, so then I started adding [to foods at home] dessert and salad. It could be a smaller portion, but as I had more things, I became used to it”* (E11)	Food availability (structure): *“I know that in the childcare center they just give them healthy foods, so I am not afraid that my child goes to the childcare center and eats because I know that they give them only good foods. They give them legumes, the same things I make here [at home]. They give them “carbonada” [a Chilean vegetable and beef soup] they give them better meat than I do…”* (E6)
Theme 2. *“They give the best foods for him/her”*	Meal and snack routines (structure): [Related to how the childcare center has influenced the child’s feeding at home] “... In the schedules... The child came in (to the childcare center) drinking from a baby bottle milk... Later “No, mom, I don’t bring the bottle”, because the child already drinks from a cup”... because almost all the children were already drinking from a cup and he wasn’t. And the other thing is that I don’t know, he [child] is one of those people who imitates everything in that aspect [of eating]... The schedule and the types of food, they do not add salt or anything... besides, they [the educators] are always asking in the childcare center if he is allergic to something [food], what he cannot eat, they always ask you to know what [food] to give him and what not to give him” (E2).	
Theme 3. *“They promote a healthy lifestyle for the whole family”*	Child involvement (autonomy support or promotion): *“In this childcare center they do help a lot…, the projects they have done here, they have done one of healthy lifestyle, last year they had physical education teachers, they had zumba lessons for mums... Now these days they ask for fruit every day in the classroom because I think that during recess when children are very restless, they tell them “ok, go get a fruit”… Sometimes, any day of the week they do healthy activities, say, making an “alfajor” but a healthy one, or I don’t know, a fruit salad, that the children themselves make, so [the child] likes that”* (E21)	Nutrition education (autonomy support or promotion): *“In the childcare center [the child learnt about front-of-package food warning labels]... There was a workshop and they did, like, activities about the labels. I mean, “don’t give me this mum because it has too many labels” [says the preschooler]... that it had too much sugar…fats, that sort of thing. The children start to, like, notice… avoid the black stuff [foods with food warning labels]”* (E14)
**Healthcare Center**		
Theme 4. *“They educate us on what to feed our child”*	Nutrition education (autonomy support or promotion): *“The nutritionist [from the healthcare center] in that sense [how to approach feeding] was very clear and she would tell me all the steps to follow, or how to do it. I told [the child] “Look, your mum is going to tell me about you… if I tell you to eat half a bread bun, it has to be half, and if not, eat a fruit.” So then they would explain everything to both, me and the child... ”* (E15)	Limited/guided choices (structure): *“The pediatrician told us that we already had to start giving some foods [offer foods to the child]. I can’t remember if there was a list of foods, yes, I think so, she gave me a list, a guide... she gave me a list of the foods I had to incorporate in the daily diet of the child, um... carbohydrate from potato, pasta or rice plus a protein, which was mostly chicken, and then to start with a certain amount of vegetables... and that’s how we start. *(E14)
Theme 5. *“It is difficult to follow the recommendations they give us”*	Restriction (coercive control): *“… I have nothing to say, they have explained me well [in the public healthcare center about the preschooler’s feeding]. The problem is that sometimes, how do you do it? Because I say, “ok, if they give the child milk at 4 and at 7 because they should eat every three hours, she* [*the nutritionist*] *says to give him a little amount, I mean, one tries, really, but I told her: he [the child] really likes eating fruit. She* [*the nutritionist*] *said, “... fruit is healthy but in big amounts it is also harmful,” so, then what do I give him?”* (E15)	Restriction (coercive control): *“I tell the nutritionist [at the healthcare center] yes, yes, but then, I still give [the child] some foods, because then what is wrong with yogurt, jelly? As I am saying, for her everything is bad, [the child] couldn’t eat sausage, egg, or there were too many lentils... they tell me to make him gain weight, but I can’t give him everything and then if he gains weight, they are going to tell me that he should lose weight, so then it’s like, I’m done... I am not going to rack my brain... I know how he [the preschooler] is, I know what he is going to eat, and if I forbid him to eat something he is going to want to eat it even more... As I am saying, [the child] can eat many things, but just a little bit of everything”* (E7)

## Data Availability

Data are contained within the article and Supplementary Materials.

## Supplementary Materials

The following supporting information can be downloaded at: https://www.mdpi.com/article/10.3390/children10121875/s1. Supplementary File S1. Interview guidance.
