# Supplementary material for: Food Parenting Practices Promoted by Childcare and Primary Healthcare Centers in Chile: What Influences Do These Practices Have on Parents? A Qualitative Study
Source: Children (Basel). 2023 Nov 29;10(12):1875. doi: 10.3390/children10121875 (PMC10741564; doi:10.3390/children10121875)
Supplement: Supplementary file 1 [file children-10-01875-s001.zip › children-2683586-supplementary.pdf]

# Supplementary File S1: Interview Guidance

## Family/Clan factors

- What is the favorite food of your child? How do you know it?
- What are the foods that s/he does not like or like less? How do you know it? How do you help him to eat or s/he eats by himself? When a child can start eating by him/herself?
- How do you know she/he is full? How do you know when s/he is hungry?
- Are there things about feeding your child that you find fun or easy?
- Are there things about feeding your child that you find difficult to deal with?
- What goals do you have when you are feeding your child? What happens?
- Think about the times it has been difficult to feed your child. What was happening?
- What strategies do you use for feeding your child?
- Does your family or your husband's family participate in feeding your child? Do you think that your family or your husband/partner's family influence the way your child eats? When do they participate with your child (e.g., in the week or weekends)?

## Culture

- Have you heard about healthy foods and habits? How important are they important for you? How important is for your child? How did you learn to introduce new foods to your child?

- How do you perceive the weight of your child? Why?

**Community/Childcare Setting**

- Have received education or any type of instruction about how to feed your child (e.g. How to respond to your child when he/she does not want to eat)? In the childcare? or in your health care center where your child is usually attending?
- Do you know how your child is being fed in the childcare setting hours?

**Country**

- Have you seen food packages with cartoons that your child like? (e.g., cereal, yoghurt).
- Do you select any food for your child in the last year based on the food labels (notes in a black sign in the foods)?
